# Intentional internal drainage tube method for nonlocalized persistent pancreatic leakage: a case report

**DOI:** 10.1186/s12893-021-01188-1

**Published:** 2021-04-19

**Authors:** Kinji Furuya, Tatsuya Oda, Osamu Shimomura, Yusuke Ozawa, Kenichi Iwasaki, Yoshihiro Miyazaki, Manami Doi, Koichi Ogawa, Yohei Owada, Yusuke Ohara, Kazuhiro Takahashi, Yoshimasa Akashi, Katsuji Hisakura, Tsuyoshi Enomoto, Jaejeong Kim, Shinji Hashimoto

**Affiliations:** Department of Gastrointestinal and Hepato-Biliary-Pancreatic Surgery, Faculty of Medicine, University of Tsukuba, 2-1-1, Amakubo, Tsukuba-shi, 305-8575 Ibaraki Japan

**Keywords:** Pancreatic leakage, Disconnected pancreatic duct syndrome, Internal drainage, External drainage, Surgery, Systemic inflammatory response syndrome, Case report

## Abstract

**Background:**

Persistent pancreatic leakage (PL) due to disconnected pancreatic duct syndrome (DPDS) is associated with severe morbidity and mortality and it usually treated with internal drainage. However, in cases without localized fistula formation, internal drainage is challenging to perform. We report an original one-stage surgical approach for nonlocalized persistent PL, namely, the “intentional internal drainage tube method”.

**Case presentation:**

A 49-year-old woman whose main pancreatic duct was penetrated during endoscopic retrograde cholangiopancreatography experienced severe PL. Peritoneal lavage and a second operation involving central pancreatectomy failed to relieve the symptoms, and nonlocalized PL persisted due to DPDS. Although we attempted a radical resection of the pancreatic remnants as a third strategy, the highly inflamed tissue and massive bleeding prevented the completion of the procedure. We sutured the pancreatic head margin and performed a pancreaticojejunostomy to the distal margin. Because these two cut margins could possibly be the source of the persistent PL, we created a hole at the Roux-en-Y jejunal limb, and a silicone drainage tube was inserted into the peritoneal space via this hole. Postoperatively, we continuously suctioned the intentional internal drainage tube, and the residual PL cavity gradually diminished. Even after removal of the tube, the residual PL drained internally into the jejunum through this hole.

**Conclusions:**

We present this intentional internal drainage tube method as a novel alternative approach for the management of nonlocalized PL consequential of DPDS. Due to the simplicity and minimally invasive nature of this method, we propose this technique may also be used to treat various types of nonlocalized persistent PL or be used prophylactically for central pancreatectomy.

## Background

Pancreatic leakage (PL) is a complication associated with various pathophysiological conditions, such as pancreatitis, surgery, and trauma, including iatrogenic events from invasive medical procedures such as endoscopic retrograde cholangiopancreatography (ERCP) [1–5]. If PL persists, autodigestion of peripancreatic tissues occurs, which can cause ruptured aneurysms and/or multiple organ failure [1, 2, 6–8]. One cause of severe PL is disconnected pancreatic duct syndrome (DPDS). DPDS is characterized by blockage of the main pancreatic duct (MPD) with no access to the upstream pancreatic duct, concurrent with a persistent nonhealing pancreatic fistula or pancreatic fluid collection [3–5, 9, 10].

In cases of severe DPDS, patients may develop systemic inflammatory response syndrome (SIRS) with nonlocalized PL, and two therapeutic strategies are often reluctantly attempted. One approach is resection of the damaged pancreas, which is complicated by the extensive tissue inflammation. The other approach is a two-stage surgery, comprising excessive external drainage with peritoneal lavage to generate a rigid pancreatic fistula and an additional surgery to convert the fistula into an internal drainage route, which is complicated by the development of adhesions [1, 2, 5, 9, 11].

We report the successful treatment of nonlocalized persistent PL in a patient in whom radical resection was not possible. Our original approach is termed the “intentional internal drainage tube method”, which involves the insertion of a trans-jejunal external drainage tube. The application of this strategy resulted in the successful recovery of the patient from severe post-ERCP pancreatitis.

## Case presentation

**Fig. 1:**
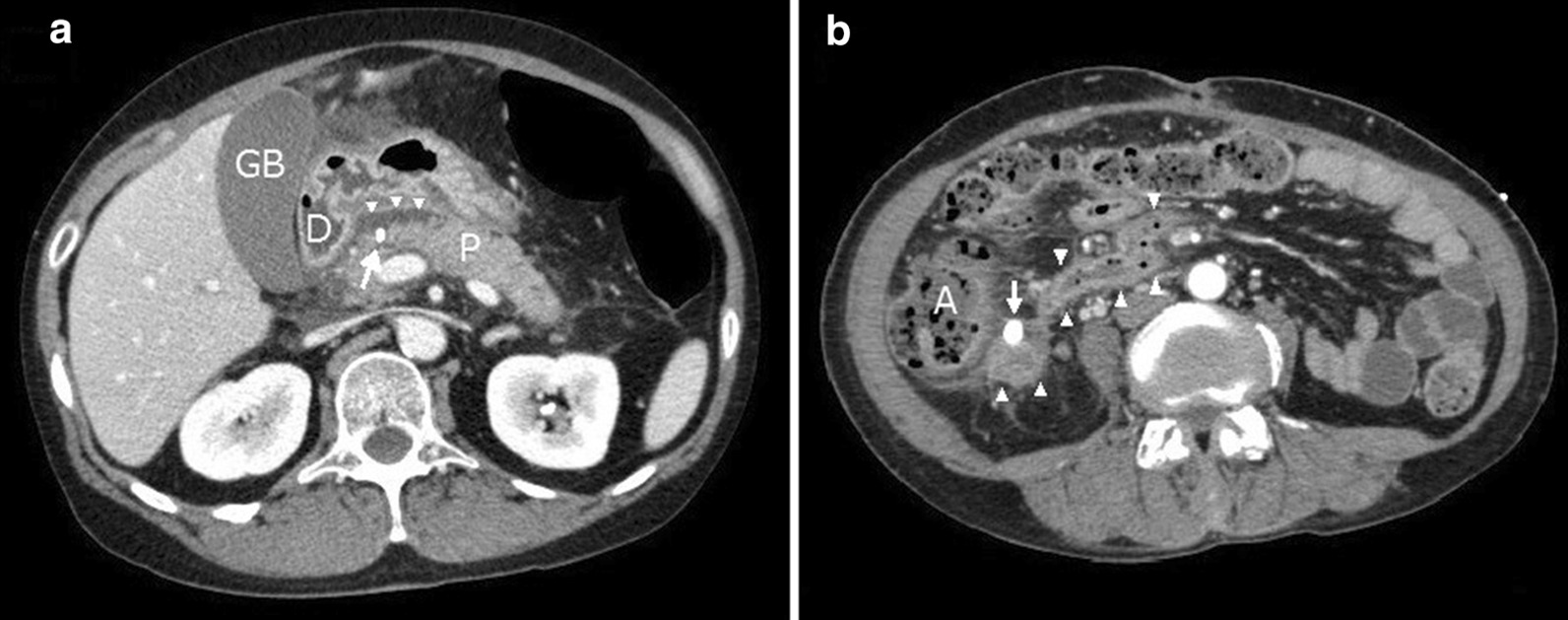
CT scans of the initial damage to the MPD and prolonged pancreatic leakage. **a** POD 1. The pancreatic duct stent tube is dislocated and protruding into the peritoneal cavity (arrow), and fluid collection over the pancreatic surface (arrowhead) is evident. **b** POD 80, before the third operation. Pancreatic fluid surrounded with inflamed tissue spread toward lower peritoneal space (arrowhead). A drainage tube (arrow) was placed near that cavity. *GB* gallbladder, *D* duodenum, *P* pancreas, *A* ascending colon

A 49-year-old asymptomatic woman underwent ERCP to investigate asymptomatic focal dilatation of the MPD. During ERCP, a guidewire possibly penetrated the pancreatic parenchyma. Although a prophylactic pancreatic duct stent was placed at the end of the procedure, the patient developed post-ERCP pancreatitis on postoperative day (POD) 1, as demonstrated by upper abdominal pain, fever, and a high serum amylase level (1959 U/L). A contrast-enhanced computed tomography (CT) scan revealed peripancreatic fluid collection, and the pancreatic stent tube was dislocated in the peritoneal cavity at the neck of the pancreas (Fig. 1a). We speculated that this dislocated tube injured the MPD and induced massive PL. On POD 2, we attempted to replace the pancreatic stent tube to bridge the damaged point of the MPD via an endoscopic approach. Even after successful bridging of the MPD stent tube, the nonlocalized PL worsened, and the patient developed SIRS and acute respiratory distress syndrome (ARDS).

On POD 3, an open laparotomy was performed for the peritoneal lavage. The MPD penetration point could not be visualized because the peripancreatic tissue was severely damaged. We completed the procedure by placement of 12 external drainage tubes. Although continuous peritoneal lavage was performed, SIRS worsened and became life-threatening, and fluid with a high amylase concentration (> 30,000 U/L) spread to the right side of the peritoneal space.

**Fig. 2:**
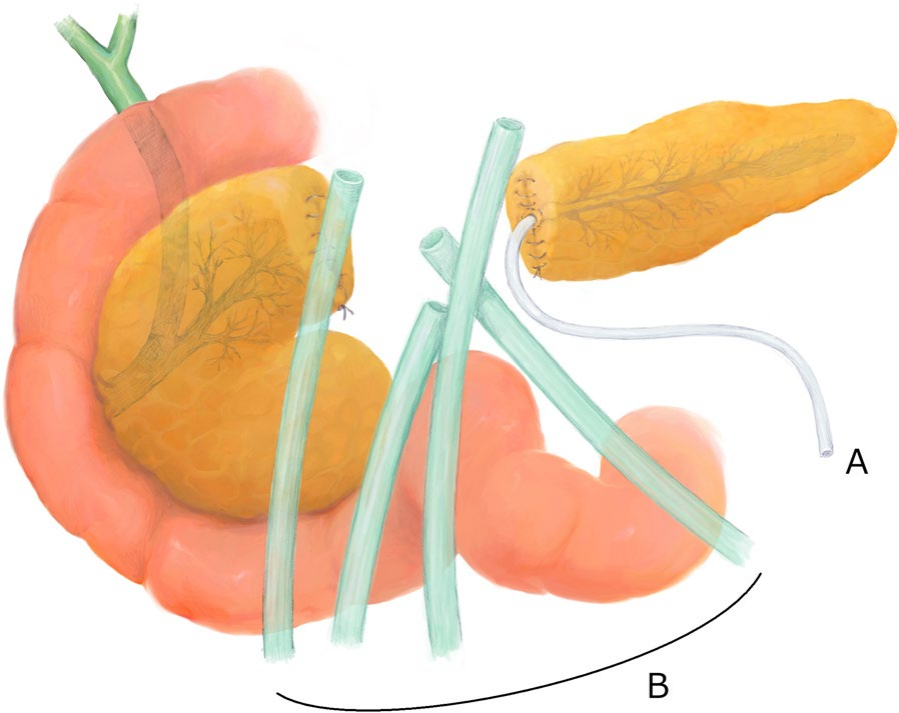
Schematic of central pancreatectomy during the second surgery. The severely damaged central part of the pancreas was resected. An external drainage tube (**A**) was inserted into the distal MPD. An external drainage tube (**B**) was placed

We then performed a second operation on POD 9, which involved central pancreatectomy with necrosectomy (Fig. 2). The proximal incision margin was closed using manual sutures. The damaged distal surface was fragile; therefore, only an external drainage tube was inserted into the distal MPD instead of performing pancreatojejunostomy. At the end of the surgery, five external drainage tubes were placed around the two damaged parenchymal surfaces. After the second operation, the patient’s SIRS showed immediate improvement; however, the PL was still not controlled. On POD 70, the patient again developed SIRS and complained of severe abdominal pain. Contrast-enhanced CT revealed pancreatic fluid spread throughout the retroperitoneal space (Fig. 1b).

**Fig. 3:**
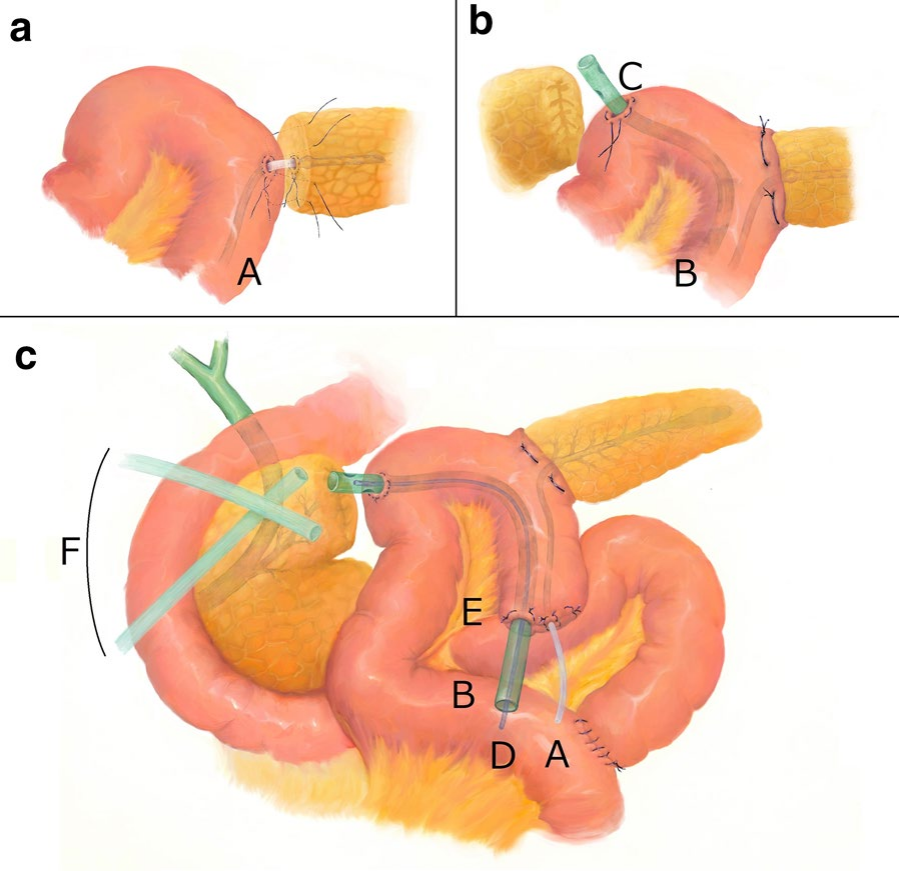
Schematic demonstration of the “intentional internal drainage tube method” applied in the third surgery. **a** The distal pancreas was anastomosed to the elevated R-Y jejunal limb with an external MPD stent tube (A). **b** A single-lumen silicone drainage tube (B) was placed through the jejunal hole such that the tip of the tube was located in the peritoneal cavity (C). This hole was occupied with tube B, the “intentional internal drainage tube,” by the tobacco suture. **c** A thin tube for continuous suction (D) was inserted in tube B. The jejunal blind stump (E) was fixed to the abdominal wall and drained into tubes A, B, and D outside of the body without running through the peritoneal cavity. Extra external drainage tubes (F) were placed

A third surgery was scheduled on POD 80 to remove the remnant pancreas, which might have caused the PL due to DPDS. Laparotomy showed that both edges of the pancreatic remnants were heavily damaged; thus, we could not determine which edge was the origin of the PL. We attempted to remove both remnants; however, the highly inflamed tissue and collateral venous plexus made the procedure difficult and finally led to massive bleeding from the superior mesenteric-portal vein confluence. We terminated pancreatic resection and performed pancreatojejunostomy. The distal MPD and pancreatic parenchyma were anastomosed to the Roux-en-Y (R-Y) jejunal limb with an external pancreatic duct stent (Fig. 3a). Additionally, we closed the proximal MPD and the pancreatic surface using manual sutures. We were not confident in the success of either procedure because both surfaces were severely damaged and fragile; thus, the recurrence of persistent leakage was highly suspected.

Consequently, we decided to generate an intentional internal drainage route during the operation (Fig. 3b). This “intentional internal drainage tube method” was designed to collect possible persistent PL from both margins of the pancreas. A 22-Fr single-lumen silicone drainage tube (Fuji systems Co., Tokyo, Japan) was placed in a trans-jejunal orientation. The tip of the tube was located in the peritoneal cavity to collect the possible PL from both sides. The middle part of the tube ran along the inner lumen of the R-Y jejunal limb, and the distal end protruded toward the outside of the body (Fig. 3c). The jejunal fistula, through which the tube passed, was tied with an absorbable tobacco suture to prevent jejunal fluid from leaking into the peritoneal space.

**Fig. 4:**
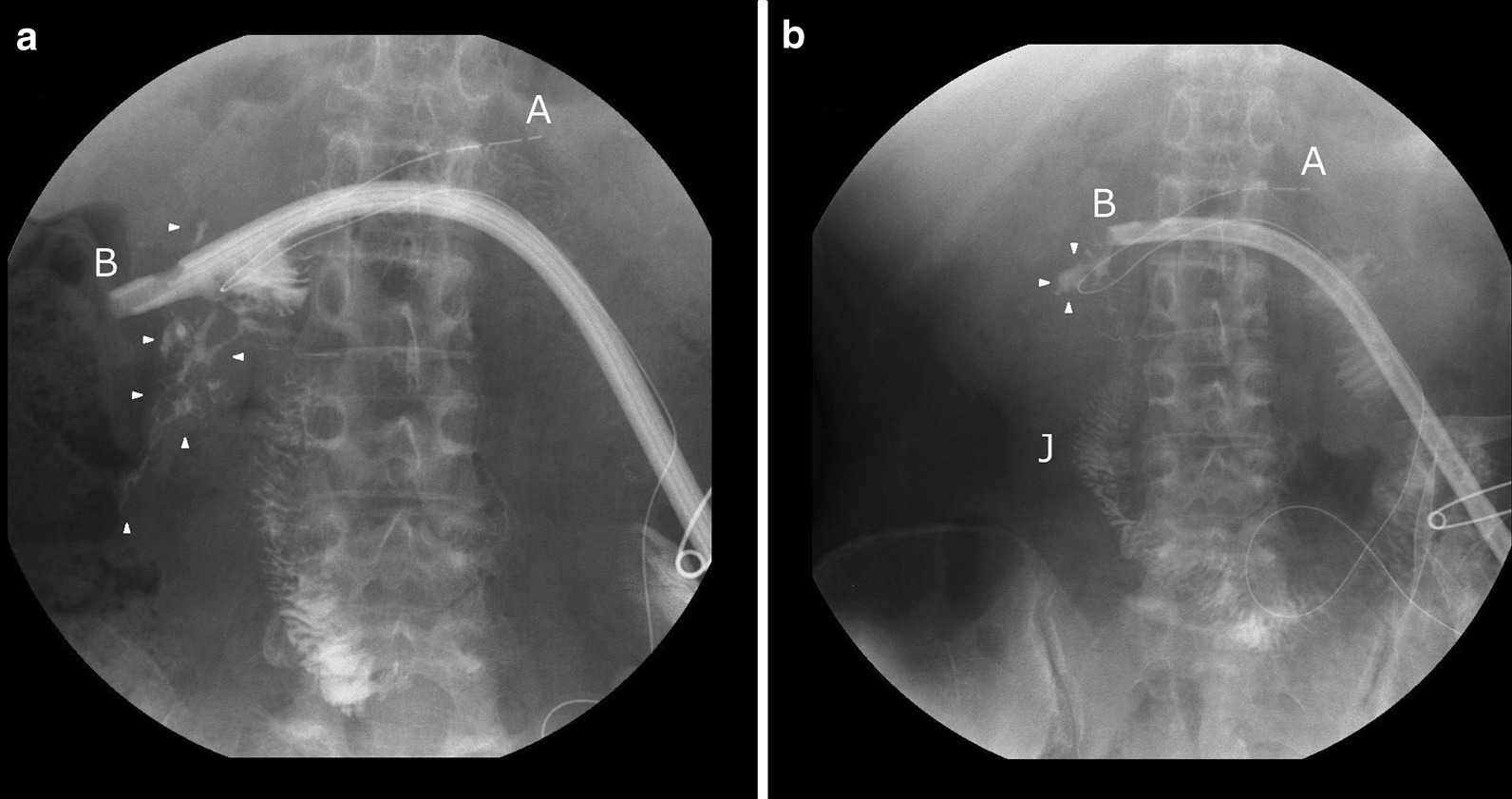
Fistulography from the intentional internal drainage tube. **a** POD 124. Fistulography of the intentional internal drainage tube revealed the localized cavity of the PL (arrowhead). Most of the contrast medium smoothly flowed into the R-Y jejunal limb. **b** POD 130. Only a small peritoneal cavity was identified (arrowhead), demonstrating the completion of internal drainage. **a** The pancreatic duct stent tube was inserted into the distal pancreas (as in Fig. 3). **b** The trans-jejunal drainage tube (same as Fig. 3). J: R-Y trans-jejunal limb

Immediately after surgery, we connected a thin suction tube inside this intentional internal drainage tube to a continuous suction device to collect PL mainly from this tube. The device was set up for periodic suction with a pressure of − 30 cm (water column) to avoid tissue injury. Necrosed deposits were effectively excreted from this “intentional internal drainage tube”, and we continued peritoneal lavage around the pancreas with saline for more than 3 weeks. SIRS gradually resolved in the patient. Within 2 months after the third surgery, we gradually removed the peripancreatic external drainage tubes, and only the intentional internal drainage tube remained. Repeated contrast radiography of the intentional internal drainage tube demonstrated that the PL cavity gradually localized and decreased in size (Fig. 4). On POD 140 after the initial ERCP, we verified that the PL was localized to a size of 2 × 1 cm and removed the intentional internal drainage tube. Thus, an internal drainage route, i.e., communication between the PL and R-Y jejunal limb, was established. The patient was discharged on POD 147 after the initial ERCP. Over the subsequent 5 years to date, the patient has recovered well without any further hospitalization and has resumed her healthy social life.

## Discussion and conclusions

We present here our original “intentional internal drainage tube method”, resulting in the successful recovery from persistent PL due to DPDS. The tube was initially intended to drain pancreatic deposits to the outside of the body. After removal of this tube, the fistula in the jejunal wall served as an internal drainage route to transfer pancreatic deposits into the intestinal lumen. Once a mature internal drainage route is generated, relapse or complications should be rare, as has been reported with endoscopic and/or surgical approaches for internal drainage [2, 5, 12]. In fact, our patient has recovered uneventfully for over 5 years.

In the third surgery in this case, resection of the pancreatic remnants was found to be difficult. Therefore, only internal drainage remained as possibility for radical treatment. As a treatment for DPDS, two-stage surgery is an established method of internal drainage [4, 5, 10, 13]. In this case, the formation of a localized rigid drainage route was essential; this formation depends on the balance among the amount of leakage, the amount of drainage, and the patient’s wound healing potential. Previous reports mentioned that longer than 4 weeks is needed for the establishment of a rigid drainage route [10]. Thus, this conventional approach necessitates the long-term placement of an external drainage tube, which decreases the patient quality of life. In addition, it was not clear that the fistula and PL cavity would be localized after such a long period. We had already failed to localize the pancreatic fistula and were concerned that the patient’s healing potential might become substantially reduced during a long hospitalization.

There are few guidelines detailing treatment strategies for DPDS. The American Gastroenterological Association recommends distal pancreatectomy in the first 30–60 days for DPDS, but this approach has high morbidity [14]. The European Society of Gastrointestinal Endoscopy guidelines recommend long-term placement of transluminal plastic stents after transluminal drainage of walled-off necrosis in DPDS patients [15]. If the endoscopy approach fails, surgery including distal pancreatectomy or R-Y drainage is recommended. The Italian and Japanese guidelines do not specifically mention DPDS, but they state that intervention (including radiological, endoscopic, or surgical) should be performed when necrotizing pancreatitis leads to clinical deterioration or ongoing organ failure [16, 17].

As Yamada et al. reported, endoscopic ultrasonography-guided drainage is one option for DPDS [18]. It is less invasive than two-stage surgery. However, the success rate has been reported as ranging from 38 to 73% [3, 19]. The difficulty of this procedure depends on the anatomical condition, which is impossible to predict in advance. Moreover, this method is not applicable in all cases. In our method, we were able to anatomically design the internal drainage route prior to the procedure. This approach is relatively easy and feasible.

An experienced surgeon may be concerned that the fistula generated in the jejunum via our “intentional internal drainage tube method” may worsen PL due to the reverse overflow of bacteria-rich intestinal juice, thereby activating the pancreatic juice. This drawback has been overcome as follows. First, the jejunal fistula was designed at the R-Y limb, where bile and food do not pass through. Second, the created fistula only contained the tube held by a tobacco suture. Third, immediately after surgery, the intentional internal drainage tube was connected to a continuous suction device. This suction device mainly served to direct PL into this intentional internal drainage tube rather than into the other external drainage tubes. These maneuvers aimed to prevent the reverse flow of contaminated intestinal juice into the peritoneal cavity.

We applied this intentional internal drainage tube method during the third operation on day 80. However, after reviewing the case history, we noted that it could also have been performed in the second operation on POD 9 to shorten the hospital stay. Due to the simplicity and minimally invasive nature of this method, we believe this new method may represent an alternative approach for treating various types of nonlocalized persistent PL and may also be used prophylactically for central pancreatectomy in which there are two dangerous causative margins for severe PL.

## Data Availability

Data sharing is not applicable to this article, as no datasets were generated or analyzed during the current study.

## Abbreviations

PL: Pancreatic leakage
ERCP: Endoscopic retrograde cholangiopancreatography
DPDS: Disconnected pancreatic duct syndrome
MPD: Main pancreatic duct
SIRS: Systemic inflammatory response syndrome
POD: Postoperative day
CT: Computed tomography
ARDS: Acute respiratory distress syndrome
R-Y: Roux-en-Y
